# Relationships between the Spinal Dural Pulsations and the Short-Term Efficacy of Lumbar Epidural Steroid Injection

**DOI:** 10.1155/2024/1824269

**Published:** 2024-03-18

**Authors:** Chan Hong Park, Sang Ho Lee

**Affiliations:** ^1^Department of Anesthesiology and Pain Medicine, Daegu Wooridul Spine Hospital, Daegu, Republic of Korea; ^2^Department of Neurosurgery, Chungdam Wooridul Spine Hospital, Seoul, Republic of Korea

## Abstract

**Background:**

Lumbar spinal stenosis (LSS) causes low back pain, leg pain, numbness in the leg, and neurogenic intermittent claudication. Epidural steroid injection (ESI) has been used for treating spinal stenosis symptoms. We hypothesized that dural pulsation was variable for lumbar spinal stenosis. In cases of the presence of dural pulsation, the pain relief after the ESI was better than in the absence of dural pulsation. This study aimed at investigating the relationships between the presence or absence of spinal dural pulsations and the efficacy of ESI.

**Methods:**

A total of 71 patients were enrolled in this prospective study. Prior to the ESI, the dural pulsation was measured using a 5-1 MHz array ultrasound transducer. The visual analogue scale (VAS) score was measured pre-ESI and 2 weeks post-ESI and 4 weeks post-ESI. At 4 weeks post-ESI, dural pulsation was rechecked.

**Results:**

The VAS scores improved after the ESI procedure regardless of the presence or absence of dural pulsation. There was a correlation between the pulsation of the dura and post-ESI VAS scores. However, VAS was not significantly different for different grades of stenosis.

**Conclusion:**

The ESI was effective in patients with spinal stenosis in short-term follow-up. Dural pulsation of the spinal cord was a positive predictive factor for the ESI effect, but the grade of spinal stenosis severity had no effect on the effectiveness of ESI.

## 1. Introduction

Lumbar spinal stenosis (LSS) may be caused by variable factors, with complaints such as low back pain, leg pain, numbness in the leg, and neurogenic intermittent claudication [1, 2]. Nonsurgical methods such as medication, physical therapy, and epidural steroid injection have been used to treat spinal stenosis symptoms [3, 4]. Lumbar epidural steroid injection (ESI) has commonly been used in patients with LSS and/or disc herniation [2, 5–12]. The effect of ESI is to ameliorate pain with the combined advantages of local anesthetics delivery and steroid administration. There was a previous study on the efficacy of ESI to the severity of LSS [13]. Campell et al. [14] reported that the spinal canal dimension is not predictive of the success or failure of epidural steroid injection in patients with spinal stenosis.

Recently, to increase the effect of the procedure or the accuracy of diagnosis, ultrasound has been used in the spine [15–17]. Pulsation of the spinal cord, dura mater, and cerebrospinal fluid is seen intraoperatively [18]. Additionally, periodic pulsations in the central nervous system are caused by pressure changes in the vascular system of the brain and spinal cord and can be seen by magnetic resonance imaging (MRI) [19, 20].

In general, pulsatile movements of the dura mater indicate that the cord is free within the subarachnoid space, with no extrinsic compression. Ultrasound has been used to predict surgical success for cervical laminoplasty [16]. In addition, intraoperative US has been used to evaluate the decompression status of the spinal cord in patients with cervical compressive myelopathy [21–24]. However, the presence of dural pulsation does not mean that there was no spinal cord compression and is affected by several factors [18, 21]. Furthermore, it remains unclear whether restoration of dural pulsation is associated with adequate decompression of the spinal cord, or whether dural and spinal cord motions correlate with each other [21].

We hypothesized that dural pulsation was variable depending on the lumbar spinal stenosis grade and that the presence of dural pulsation led to better pain relief after the ESI when compared to cases without pulsation. This study aimed at investigating the relationships between the presence or absence of spinal cord dural pulsations and the efficacy of ESI.

## 2. Methods

### 2.1. Study Design

A total of 71 patients were enrolled in the present study. We obtained approval from the institutional review board, and the study participants provided written informed consent.

The inclusion criteria were as follows: (1) LSS symptoms including neurogenic intermittent claudication characterized by a reduced walking distance, numbness, weakness, and discomfort in legs while walking or during prolonged standing, and regression of symptoms during rest; (2) magnetic resonance imaging (MRI)-confirmed LSS; and (3) central canal stenosis. The exclusion criteria were as follows: (1) symptoms unclear, (2) lateral recess or foraminal stenosis, (3) spondylolisthesis, (4) previous back surgery, and (5) bleeding tendency.

The LSS grade followed the new grading system [25]. Prior to the procedure, the dural pulsation was measured using a 5-1 MHz array ultrasound transducer (CX50®, Philips, CA, USA), and the B-mode was checked in the prone position (whether present or not). Pulsatile movements of the spinal cord and dura mater were checked at the narrowest level of the lumbar canal, which was identified on preoperative MRI and by transverse and longitudinal transducer orientations to the spinal canal. After confirmation of dural pulsation, the information was recorded.

The ESIs were performed with a CT unit (Big Bore, Philips, USA). Patients were prepared on the radiological table in the prone position, and a wire marking device wire was placed at a suitable location on the low back, and scanning of the pelvis was acquired at 120 kV and 60 mA; the slice diameter and index were 1 mm. Scans were acquired of the undersurface of the posterior lumbar spinous process. From this view, the distance between the introduction angle and the epidural space was measured at the same level of lesion for all subjects. After marking the skin at the appropriate spot near the midline, the area was sterilized and anesthetized.

A 22-gauge Tuohy needle was then advanced partially into the patient, and the needle was then advanced downward onto the outer aspect of the ligamentum flavum by using intermittent CT guidance. After placing the needle in the epidural space using the loss-of-resistance technique, 0.5 ml of contrast agent was injected to confirm the epidural space. A combination of 2 ml of preservative-free 1% lidocaine, 1500 units of hyaluronidase, and 4 mg of dexamethasone (1 ml), for a total volume of 3–5 ml, was injected into the epidural space.

The visual analogue scale (VAS) score was measured preoperatively and 2 weeks and 4 weeks after ESI. Additionally, postprocedure pulsation was measured at four-week postprocedure follow-up. The VAS score was evaluated by using the chi-squared test. Correlations between pain relief and the presence or absence of dural pulsation were evaluated using Spearman's rank correlation test. Statistical significance was set at *P* < 0.05.

## 3. Results

A total of 71 patients including 27 men and 44 women (aged 50–89 years; mean 68.2 years) diagnosed with central LSS were included in the study. There was no significant difference between dural pulsation presence group and absence group in age, gender ratio, frequency of stenosis level, and stenosis grade. The levels and sites of the affected regions are shown in Table 1: L4/5 was the most frequently implicated region.

The VAS score was improved after ESI, regardless of the presence or absence of dural pulsation (Table 2). There was a correlation between the pulsation of the dura and the ESI effect (Table 2). In cases of the presence of dural pulsation, VAS score significantly reduced than in the absence of dural pulsation group. According to the grade of stenosis, 41 patients (57%) had grade 1 stenosis. Four weeks after the procedure, the VAS score was not significantly different for different grades of stenosis (Table 3).

None of the cases switched from having dural pulsation to having it or vice versa two weeks after the procedure.

## 4. Discussion

In our study, the dural pulsation did correlate with the effect of ESI. However, the grade of spinal stenosis had no effect on the effectiveness of ESI. Our results were not consistent with previous study [13, 16, 21].

The movement of the dura originates from the pulsation of the posterior spinal artery and produces a low-grade amplitude periodic motion [18]. Stenosis of the spinal canal and compression of nerve structures may lead to intermittent neurogenic claudication due to congestion of the epidural venous blood and increased vascular pressure [26]. In addition, we hypothesized that neural compression by stenosis could be the cause for the disappearance of dural pulsation. The absence of dural pulsation was indicative of severe stenosis, and it might not respond well to the ESI. Dural pulsation does clearly not always indicate the absence of compression of the spinal cord [21].

In the present study, pain reduction was different from that in the absence group. The effect of ESI is to ameliorate pain with the combined advantages of local anesthetics delivery and steroids administration [27]. We thought that there would be a change in the dural pulsation after procedure. However, there was no change from absence to presence of dural pulsation or the other way. This indicates that the role of ESI may be chemical effect rather than mechanical decompression. Also, dural pulsation does clearly not always indicate the absence of compression of the spinal cord [21]. The presence of spinal pulsation indicates low tonicity in the spinal cord and favorable circulation in the radicular arteries [28].

In our study, the B-mode US was used. We attempted to determine the dural pulsation easily and efficiently. The M-mode or another mode was applied to lumbar spine in our study. In our study, B-mode was the best modality to find dural pulsation in the epidural space. However, further studies are required to determine which mode is the most efficient.

The present study has several limitations. The first is the short initial follow-up period. Second, we did not study the degree of pulsation strength, such as absence, weak, fair, and strong. Third, the outcome was measured only by the patient's pain score; there was not a functional outcome measurement or measurement of psychological improvement, medication reduction, or disability status. Fourth, multiple level LSS was excluded, although most patients do have more than one level of stenosis. In addition, we have not ruled out comorbid diseases such as cardiac diseases, atherosclerosis, or diabetes mellitus as other decreasing cause of dural pulsation. Finally, this study did not include a variety of other comorbid diseases, mobilization level, exercise level, or activities of daily living, etc., in two groups.

In conclusion, ESI was effective for patients with spinal stenosis. Dural pulsation of the spinal cord could be a positive predictive factor for the ESI effect; however, the severity of spinal stenosis had no effect on the effectiveness of ESI.

## Figures and Tables

**Table 1:** Patient's characteristics.

*N* = 71	Dural pulsation	
	Presence (*n* = 31)	Absence (*n* = 40)
Age (yrs)	65.7 ± 11.0	70.7 ± 8.5
Gender (M : F)	10 : 21	17 : 23
Stenosis grade		
Grade 1	21 (66.7%)	20 (50%)
Grade 2	8 (26.7%)	10 (25%)
Grade 3	2 (6.7%)	10 (25%)
Stenosis level		
L23		2 (6.3)
L34	4 (13.3)	10 (25%)
L45	21 (66.7%)	23 (56.3)
L5/S1	6 (20.0)	5 (12.5%)

**Table 2:** Correlation between the presence or absence of dural pulsation and postprocedure visual analogue scale (VAS) score evaluated by using the chi-squared test.

	Presence of pulsation (*n* = 31)	Absence of pulsation (*n* = 40)	*P* value
Pre-VAS	7.3 ± 0.5	7.3 ± 0.5	0.901
Post-VAS (2 weeks)	3.3 ± 0.4	4.3 ± 1.7	0.03
Post-VAS (1 month)	3.2 ± 0.6	4.3 ± 1.9	0.03
*P* value	0.002	0.002	

**Table 3:** Correlation between the grade of spinal stenosis and postprocedure visual analogue scale (VAS) score evaluated by using Pearson coefficient.

Stenosis (*N* = 71)	Pre-VAS	Post-VAS (2 weeks)	Post-VAS (1 month)
Grade 1 (*n* = 41)	7.3 ± 0.5	3.7 ± 1.6	3.6 ± 1.6
Grade 2 (*n* = 18)	7.0 ± 0.2	3.7 ± 1.3	3.6 ± 1.7
Grade 3 (*n* = 12)	7.8 ± 0.4	4.4 ± 1.9	4.5 ± 0.9
*P* value		0.406 (*r* = −0.158)	0.909 (*r* = 0.254)

## Data Availability

The data used to support the findings of this study are available from the corresponding author upon request.
